# Spatio-temporal correlation networks of dengue in the state of Bahia

**DOI:** 10.1186/1471-2458-14-1085

**Published:** 2014-10-18

**Authors:** Hugo Saba, Vera C Vale, Marcelo A Moret, José Garcia V Miranda

**Affiliations:** Universidade do Estado da Bahia, Salvador, Bahia Brasil; Senai/Cimatec, Salvador, Bahia Brasil; Physics Institute - Universidade Federal da Bahia, Salvador, Brasil

**Keywords:** Dengue, Correlation, Transport, Randomization, Bahia

## Abstract

**Background:**

Dengue is a public health problem that presents complexity in its dissemination. The physical means of spreading and the dynamics of the spread between municipalities need to be analyzed to guide effective public policies to combat this problem.

**Methods:**

This study uses timing varying graph methods (TVG) to construct a correlation network between occurrences of reported cases of dengue between cities in the state of Bahia-Brazil. The topological network indices of all cities were correlated with dengue incidence using Spearman correlation. A randomization test was used to estimate the significance value of the correlation.

**Results:**

The correlation network presented a complex behavior with a heavy-tail distribution of the network edges weight. The randomization test exhibit a significant correlation (P < 0.0001) between the degree of each municipality in the network and the incidence of dengue in each municipality.

**Conclusions:**

The hypothesis of the existence of a correlation between the occurrences of reported cases of dengue between different municipalities in the state of Bahia was validated. The significant correlation between the node degree and incidence, indicates that municipalities with high incidence are also responsible for the spread of the disease in the state. The method proposed suggests a new tool in epidemiological control strategy.

## Background

Dengue is a tropical disease of viral origin transmitted through the bite of the *Aedes aegypti* mosquito. Because dengue is an important arboviral disease, it is important to clearly define which control objectives can in fact be achieved and which preventive measures are required to do so. Dengue has a higher incidence in tropical countries where the climate is favorable to the proliferation of the *A aegypti* mosquito. In 2012, there were approximately 2.5 billion people worldwide at risk of infection. As a result, dengue is considered one of the most serious public health problems among reemerging diseases [1].

Two-fifths of the world' population is at risk of dengue infection. The lack of effective drugs and vaccines makes vector control the sole tool for primary intervention. Understanding the dengue virus, the transmitting agent, and its interactions with the host is essential for the development of epidemiological control strategies [2, 3].

Many factors are responsible for the resurgence of epidemics of classic and hemorrhagic dengue in the last years of the 20th century. Demographic and social changes, such as population growth, urbanization, and modern transport, contribute to the increased incidence and geographic spread of dengue. The prevalence of this public health problem is greater in tropical areas of Africa, Asia and the Americas because the vector does not survive at low temperatures. The epidemiological situation in Latin America is similar to the situation in Southeast Asia, where multiple serotypes circulate, thus leading to an increased number of cases of classic and hemorrhagic dengue. In 2002, Latin American countries reported more than one million cases of dengue, among which approximately 17,000 cases were of hemorrhagic dengue, which resulted in 225 deaths [4, 5]. Dengue is a major cause of morbidity in the tropics, especially prior to 1999 [6, 7].

Despite being a disease with significant impact on public health policies, there are unanswered questions about dengue’s spreading dynamics, such as the importance of the means of transport or the network of disease spreading across municipalities.

This work aims to study the mechanisms for the spread of dengue across municipalities in the state of Bahia - Brazil. We used an adapted version of the correlation network method proposed by Eguiluz et al. [8] to build the relationships between municipalities.

Correlation networks have been used to characterize various dissemination processes in complex systems, such as seismic events in California [9], rainfall indices [10], firing rates in neurons [11, 12], brain activity [8, 13], climatic factors [14], and other factors related to dengue [15, 16]. In all of them, the generated networks provided insight into new properties due to the complex structure of the interactions among network elements.

In this study, we evaluated the hypothesis that there are correlations between the occurrences of cases of dengue between municipalities in the state of Bahia and that the network generated from these correlation is related to the information on the mechanism of disease spread in the state.

## Methods

To assess temporal trends in connectivity between cases of dengue, we used the mathematical basis of the time-varying graph (TVG) formalism [17–19].

### Time-varying graphs (TVG)

According to the conventional formalism of graph theory, a graph is defined as G (E, V), where V represents the set of vertices or nodes and E the set of edges e_i,j_, with i and j ∈V such that E ⊆ V × V, i.e., each edge of the set E is defined by the ordered pair (V_i_, V_j_). Because a TVG is a dynamic system, the relationship between its elements is considered in a defined time interval T ⊆ N, where T represents the lifetime of the system, and Ν is the set of natural numbers. Thus, the TVG formalism in its simplified form can be defined as a graph G (V, E, F), where F represents the edge presence function, F: E × T ➔ {0, 1}. This function indicates whether there is an edge e_i,j_ ∈E at a given time t ∈T.

### Time-varying correlation networks (TVCN)

The use of the correlation between time series to build networks was originally proposed by Eguiluz et al. [8] in studies on brain activity performed with a functional magnetic resonance imaging device. In that study, the different brain regions (voxels) represented the vertices of the network, and the occurrence of significant correlations between the time series of activity represented the edges of the network. A generalization of the method developed by Eguiluz et al. [8] was proposed by Silva et al. [12]. In the proposed method, the correlations between nodes are calculated only for a time window smaller than the size of the series. Based on this modification, the authors estimated the temporal evolution of neuronal activity networks in mice by sliding the window along the neuronal firing time series.

The method proposed in this paper adds the concept of static aggregated networks (SAN) to the method proposed by Silva et al. [9]. Using the formalism of the time-varying graph, we defined a graph as G(C, M), where M is the set of vertices of the network, and C is the set of edges that represents the existence of significant correlation between the time series assessed between each vertex i and j, with i and j ∈M and C ⊆ M × M. The TVCN is a dynamic system with a range defined by T and can thus be formalized as a time-varying graph G(M, C, F), where F represents the edge presence function, F: C × T ➔ {0,1}, representing the existence (1) or non-existence (0) of correlation between the time series of a pair of vertices in a given time. Another way to understand the presence function F is that it indicates the existence of an edge *C*_*i,j*_ at a given time *t* [12].

For the TVCN, this process can be formally defined as follows:
1

where *c*_*i,j*_*(t)* represents the correlation between cases of dengue in the municipalities i and j within a time window of size J centered at time t. Accordingly, this definition implies that two vertices are connected in the network only if the assessed correlation *c*_*i,j*_*(t)* is high enough (). Without loss of generality, this study assumes  to be constant over time for simplicity.

Once the set of TVG networks is created, we can integrate the networks in time so that
2

where *w*_*i,j*_ defines the weight of the edge between vertices i and j of the static aggregated network (SAN). Thus, the edges of the SAN will represent the frequency of occurrence of the edge over period T of the TVG.

### Time-varying correlation networks of cases of dengue (TVCND)

The method described in Section TVCN was applied to the daily time series of cases of dengue in 417 municipalities of the state of Bahia in Brazil for the period between 01/02/2000 and 04/16/2009. Data were collected from the database of the Notifiable Diseases Information System (Sistema de Informação de Agravos de Notificação - SINAN), an entity of the Federal Government. The SINAN database is fed by the reporting and investigation of cases of diseases and clinical conditions that appear on the national list of diseases of compulsory notification. In the creation of TVCND, the municipalities represent the vertices of the network, and their correlations in time represent the edges. The window size used was 10 days because this time is the average time for occurrence of the disease symptoms. The correlation used was the Pearson correlation, and the correlation index used was the p-value. When p-values are used to measure the correlation, the equation (1) inverts its logical expression i.e. an edge are added when .

**Figure 1:**
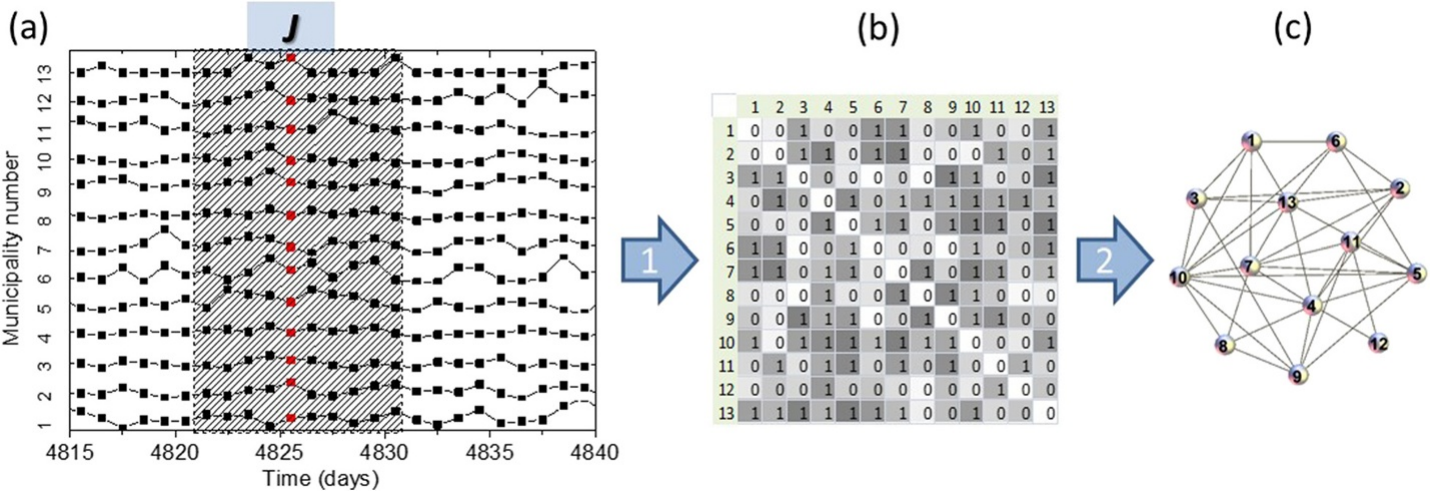
**Process of TVCND construction (Adapted from** [12]**). a)** Sliding window *J* over the time series of the municipalities set. **b)** The correlation matrix for the municipalities within the window *J*, gray color are proportional to the correlation values, a threshold is applied and the continuous correlation values are transformed in a binary matrix called adjacent matrix. **c)** Network diagram for the adjacent matrix.

In Figure 1, we illustrate the method applied to the dengue data. Figure 1(a) shows the time series for each municipality; Figure 1(b) shows the correlation matrix between all pairs of municipalities for the time window J. The grayscale matrix illustrates the different Pearson correlation values (p-value). The edge is considered for values of correlation below a critical value  and is represented by the value 1 in the adjacency matrix. It is important to remember that small p-values indicate high correlations. The network is built from the adjacency matrix in Figure 1(c).

Once a network has been built for a time t, the window is slid by a one-day increment, and a new network is calculated. The set of all networks over time forms the TVCND.

The search for the critical value of the threshold correlation  that best represents the network shows that when it is very high (represents low correlation), the noises result in the creation of many edges. Conversely, when the threshold is very low (indicating high correlations), the restrictions increase, and much information is removed from the network.

## Results and discussion

### Static aggregated networks of dengue in Bahia (SAN)

**Figure 2:**
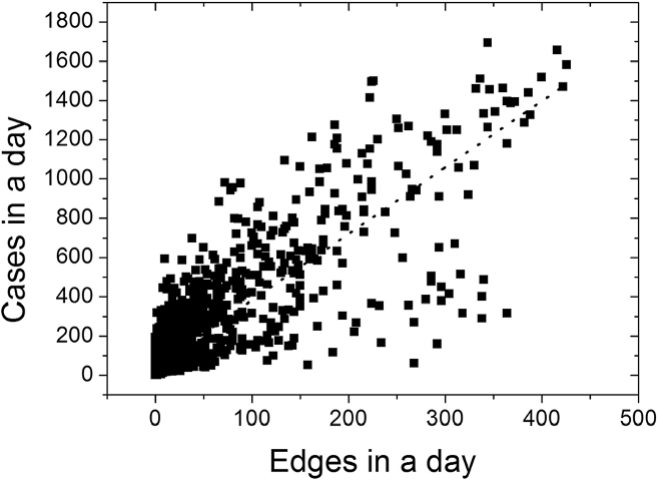
**Scatterplot between the number of edges and the number of cases of dengue per day for a network with**  **.** The fit of the data exhibited a slope of 3.38 cases per edge (P < 0.05).



The criterion used to find the optimal value of  was to adopt the value where the total number of edges of the one-day subgraph best correlates with the sum of all cases of municipalities of that day. In Figure 2, we show the scatterplot of the network generated for a presence function with the critical correlation  that exhibited the best correlation ().

This correlation shows a coupling between the number of cases in a day and network connectivity on the same day, which indicates that the connection mechanism between the municipalities is an important factor in the spread of the disease.

**Figure 3:**
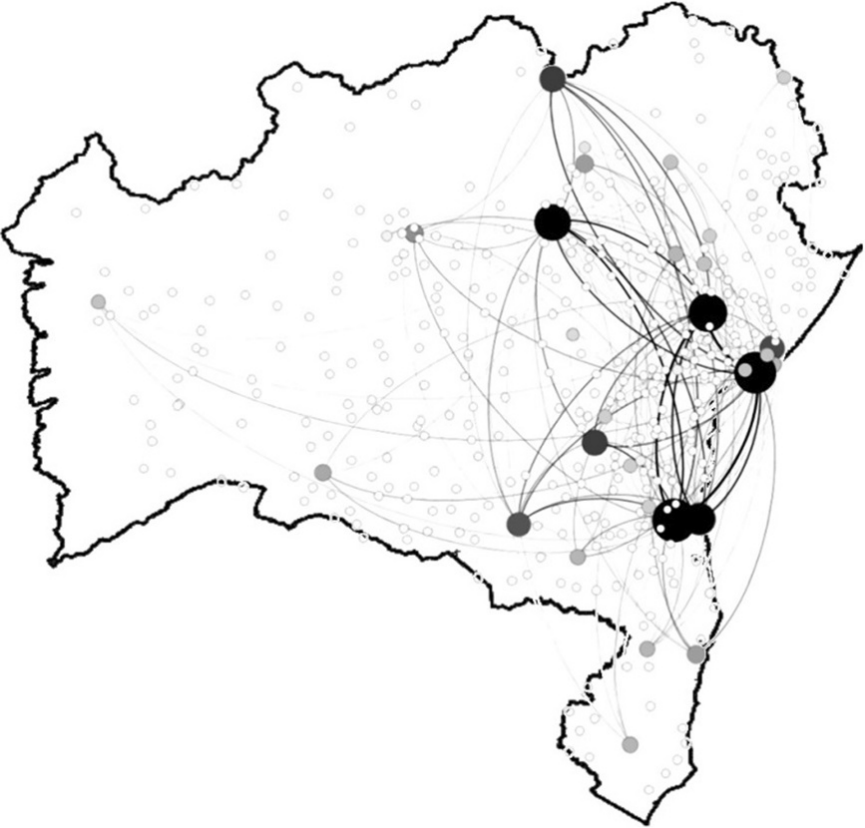
**Static Aggregated Network of time-varying correlation of dengue in the state of Bahia.** Network filtered for weights ≥100. The diameters of the vertices and the intensity of gray are proportional to the degree of each vertex.

The TVCN method was applied to cases of dengue in Bahia, generating 3393 subgraphs, where each subgraph represents a correlation network for a 10-day window of data. Its respective SAN was calculated so that a single weighted network is generated where the weights of the edges represent the number of days during which there was a correlation between adjacent vertices. Even assuming a high level of correlation (), many edges with low weight (<100 days) occur in the network. In Figure 3, we show the network for weights above 100 days. The figure shows that few nodes govern the pattern of correlation between cases of dengue in the municipalities. If the 100 days threshold filter is not used, the correlation between cases of dengue in municipalities present a great number of edges, blurring completely the figure, showing no information about the network connectivity.

We recall that if we do not use weights the correlation between cases of dengue in municipalities presents a great number of nodes featuring a highly connected graph.

**Figure 4:**
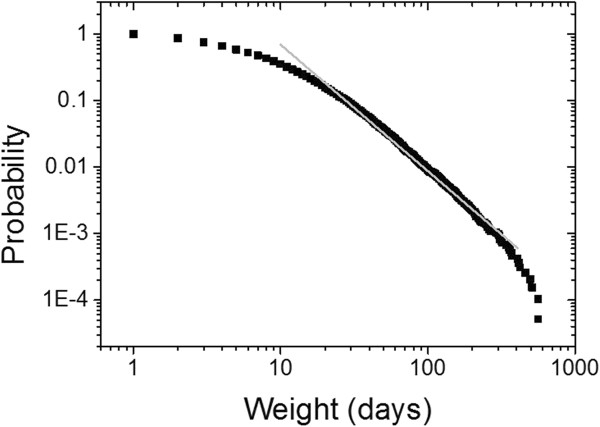
**Distribution of weights on the edges of SAN.** Linear fit in gray of the region between 10 and 365 days.

For a better evaluation of the SAN, we calculated the cumulative distribution of the weights of the edges (Figure 4). The figure shows a heavy-tailed distribution without a defined mean. The central region of the curve, between 10 and 365 days, exhibits a power-law decrease with an exponent equal to −1.90.

### Correlation between the degree of the correlation network of cases of dengue and the incidence of dengue

**Figure 5:**
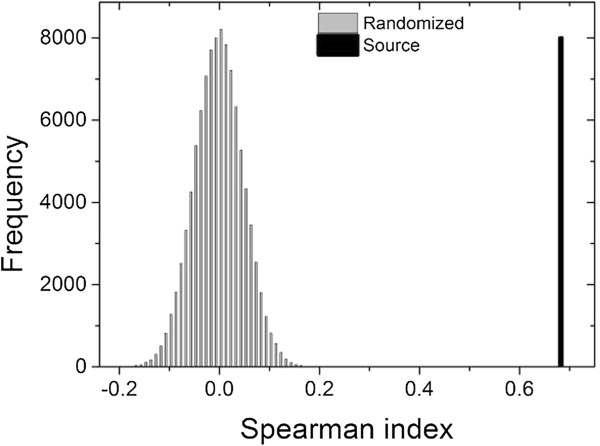
**Comparison between the distribution of Spearman correlation indices of the random set with the correlation between the degree of the municipalities in SAN and incidence of dengue in the municipalities.**

To seek an epidemiological interpretation of time-varying correlation networks of dengue (TVCND), we calculated the correlation between the degree of each municipality in the SAN and the incidence of dengue in each municipality. Using the randomization method [20–22], we applied Spearman correlation to 100,000 randomizations of the original data. The correlation was greater than or equal to the original correlation in only 0.001% of the set of randomizations, which leads to a p-value <0.00001. Figure 5 shows a comparison between the distribution of Spearman correlation indices of the random outputs with the correlation value obtained from the original data. This comparison indicates that, although low, the correlation is significant.

The existence of a significant correlation between the degree of correlation in SAN and the incidence of dengue in the municipality indicates that municipalities with high incidence are also responsible for the spread of the disease in the state.

The correlations between the occurrences of reported cases of dengue in different municipalities in the state of Bahia can aid in creating more efficient campaigns for prevention and the fight against dengue. The weights of the edges of the correlation network identify the most connected municipalities in the context of dengue in this state. In Table 1, in the case of an outbreak in the municipality of Abaíra, the municipalities of Jequié and Salvador should prioritize actions for preventing dengue.

**Table 1:** **Correlation of cases of dengue in the municipality of Abaíra**

Municipality N	Municipality M	Weight
Abaíra	Jequié	27
Abaíra	Salvador	12
Abaíra	Vitória da Conquista	7
Abaíra	Ibotirama	7
Abaíra	Fátima	7
Abaíra	Itabuna	6
Abaíra	Teixeira de Freitas	3
Abaíra	Mairi	3
Abaíra	Buerarema	1
Abaíra	Caravelas	1
Abaíra	Coaraci	1
Abaíra	Conceição do Almeida	1
Abaíra	Itajuípe	1
Abaíra	Juazeiro	1
Abaíra	Quijingue	1

## Conclusions

The correlation network of cases of dengue in Bahia shows how one municipality follows the behavior of a different municipality. The correlation between the incidence data and the correlation network itself validates the hypothesis of the existence of a correlation between the occurrences of reported cases of dengue between different municipalities in the state of Bahia.

The information generated shows municipalities with high correlation, when cases of dengue increase in a municipality, there is also an increase of cases in the other correlated municipality and vice versa. This information can guide the attention of public authorities so that when diagnosing a growth in the number of cases or even an epidemic in a municipality, campaigns can be launched in the municipalities that have the highest correlations with the municipality where the outbreak occurred. Thus, epidemics that spread through several municipalities would be minimized with regard to their spread, which would reduce its period of existence.

## Abbreviations

TVG: Timing Varying Graph
*A aegypti*: *Aedes Aegypti*
TVCN: Time-Varying Correlation Networks
TVCND: Time-Varying Correlation Networks of Cases of Dengue
SINAN: Sistema de Informação de Agravos de Notificação
SAN: Static Aggregated Networks of dengue in Bahia.
